# Adrenergic DNA damage of embryonic pluripotent cells via β2 receptor signalling

**DOI:** 10.1038/srep15950

**Published:** 2015-10-30

**Authors:** Fan Sun, Xu-Ping Ding, Shi-Min An, Ya-Bin Tang, Xin-Jie Yang, Lin Teng, Chun Zhang, Ying Shen, Hong-Zhuan Chen, Liang Zhu

**Affiliations:** 1 Department of Pharmacology and Chemical Biology, Shanghai Jiao Tong University School of Medicine, Shanghai 200025, China; 2 Shanghai Universities Collaborative Innovation Center for Translational Medicine, Shanghai 200025, China; 3 Department of Pharmacy, Renji Hospital Affiliated to Shanghai Jiao Tong University School of Medicine, Shanghai 200127, China; 4 Department of Pharmacy, Xinhua Hospital Affiliated to Shanghai Jiao Tong University School of Medicine, Shanghai 200092, China; 5 Department of Cardiology, the First College of Clinical Medical Sciences, China Three Gorges University, Hubei 443003, China

## Abstract

Embryonic pluripotent cells are sensitive to genotoxicity though they need more stringent genome integrity to avoid compromising multiple cell lineages and subsequent generations. However it remains unknown whether the cells are susceptible to adrenergic stress which can induce somatic cell genome lesion. We have revealed that adrenergic stress mediators cause DNA damage of the cells through the β2 adrenergic receptor/adenylate cyclase/cAMP/PKA signalling pathway involving an induction of intracellular reactive oxygen species (ROS) accumulation. The adrenergic stress agonists adrenaline, noradrenaline, and isoprenaline caused DNA damage and apoptosis of embryonic stem (ES) cells and embryonal carcinoma stem cells. The effects were mimicked by β2 receptor-coupled signalling molecules and abrogated by selective blockade of β2 receptors and inhibition of the receptor signalling pathway. RNA interference targeting β2 receptors of ES cells conferred the cells the ability to resist the DNA damage and apoptosis. In addition, adrenergic stimulation caused a consistent accumulation of ROS in the cells and the effect was abrogated by β2 receptor blockade; quenching of ROS reversed the induced DNA damage. This finding will improve the understanding of the stem cell regulatory physiology/pathophysiology in an adrenergic receptor subtype signalling mechanism.

The integrity of cellular DNA is challenged by various genotoxic insults. Excessive DNA damage causes mutation and genome aberrations, being toxic to the organism and predisposing to diseases such as cancer, degenerative disorders, and premature aging^1^,^2^. The genotoxic insults can be derived from endogenous sources such as reactive oxygen species (ROS) via oxidative respiration and exogenous agents like radiation and environmental/chemotherapy chemicals. Psychological stress, a sensed threat to homeostasis^3^, which we constantly experience in life, also has been reported to contribute to DNA damage, through neuronal and hormonal stress responses^4^,^5^,^6^.

The adrenergic neuronal/hormonal system is responsible for the “fight-or-flight” responses that cope with threatening or stress stimuli to the organism, by releasing adrenergic mediators adrenaline and noradrenaline that act through binding to the adrenergic receptors. Under stress conditions, the synthesis and release of the mediators are markedly augmented, physiologically or pathophysiologically, up to dozens-fold or over hundreds-fold increase in concentration^3^,^7^,^8^. The adrenergic stress has been reported to trigger DNA damage through activation of adrenergic receptors in somatic cells, implicating an important mechanism underlying the stress-related diseases^5^,^9^,^10^,^11^,^12^,^13^.

Embryonic stem (ES) cells are derived from blastocyst that is regulated by adrenergic neuronal and humoral transmission^14^,^15^,^16^. The embryonic pluripotent cells are critical for the developing embryo and are known to be susceptible to genotoxicity though they are expected to need more stringent genome integrity to avoid compromising multiple cell lineages and subsequent generations^17^,^18^,^19^,^20^. So the potential genotoxicity to the cells is raising concerns and need to be scrutinized^21^. However, it remains unknown whether the adrenergic stress mediators cause DNA damage in embryonic pluripotent cells and, if so, by which receptor signalling mechanism.

Here we have revealed that the adrenergic stress mediators lead to DNA damage and apoptosis of embryonic pluripotent cells. These effects are selectively mediated via β2 adrenergic receptor/adenylate cyclase/cAMP/PKA signalling pathway involving an induction of intracellular ROS accumulation.

## Results

### Adrenergic stimulation induces embryonic pluripotent stem cell DNA damage and apoptosis

**Figure 1:**
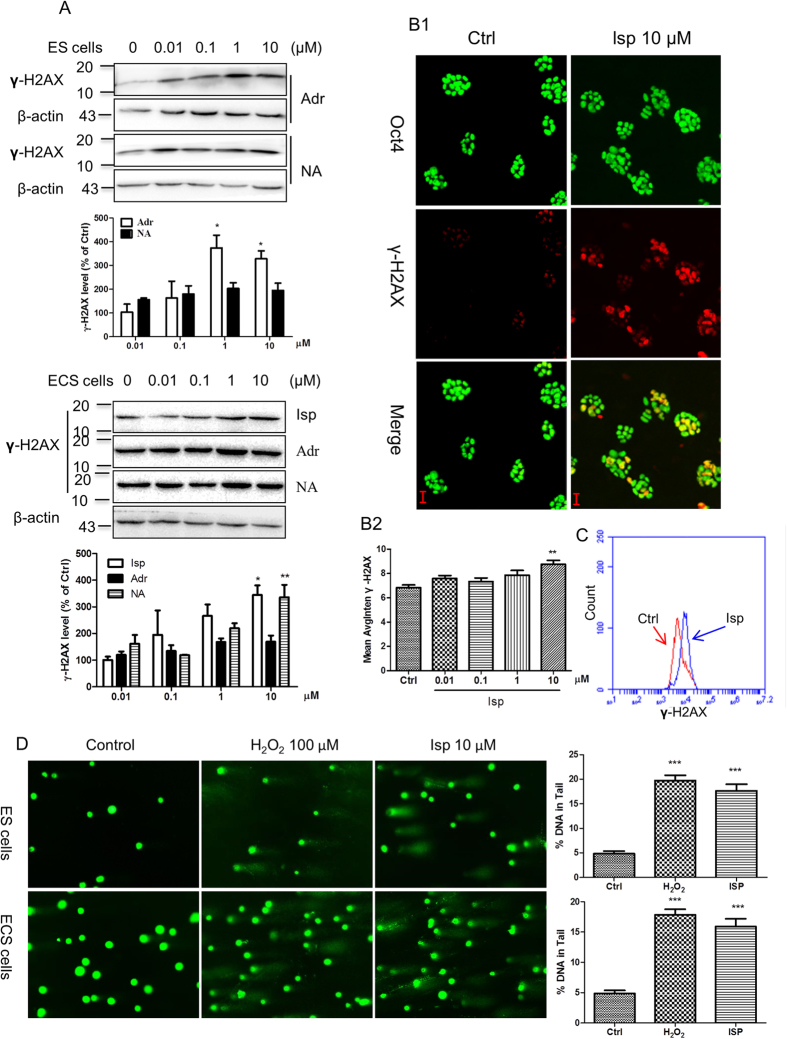
Induction of embryonic pluripotent cell DNA damage by adrenergic stimulation. (**A**) Induction of γ-H2AX accumulation in ES cells and ECS cells by adrenergic agonists assayed by western blot. Representative immunoblots are shown. Data in the column graphs show the densitometric analysis expressed as means ± SEM of three independent experiments. *p < 0.05, **p < 0.01, compared with the vehicle administrated control group. Adr, adrenaline; NA, noradrenaline; Isp, isoprenaline. ES cells, embryonic stem cells; ECS cells, embryonal carcinoma stem cells. (**B**) Induction of γ-H2AX foci accumulation by isoprenaline in ES cells imaged (B1, with 20 × magnification objective lens) and quantitatively analyzed (B2, column graph) by HCA. Oct4 is a pluripotent marker. Quantification was averaged from at least six randomly selected microscopic fields. Data represent means ± SEM from at least three independent experiments. **p < 0.01, compared with the vehicle administrated control group. HCA, High-Content Analysis. (**C**) Induction of γ-H2AX accumulation by isoprenaline in ES cells assayed by flow cytometry. Isoprenaline was used at 10 μM. (**D**) Induction of DNA breaks by isoprenaline in ES cells and ECS cells detected by comet assay (imaged with 20 × magnification objective lens). Data in the column graphs show the percentage of DNA in the comet tail expressed as means ± SEM of three independent experiments. ***p < 0.001, compared with the vehicle administrated control group. Treatment with 100 μM H_2_O_2_ for 30 min was used as the positive control for the induction of DNA damage. The cells were treated by agonists with indicated concentrations for 24 h unless otherwise indicated. The gels have been run under the same experimental conditions. Full-length blots are presented in Supplementary Fig. S6.

**Figure 2:**
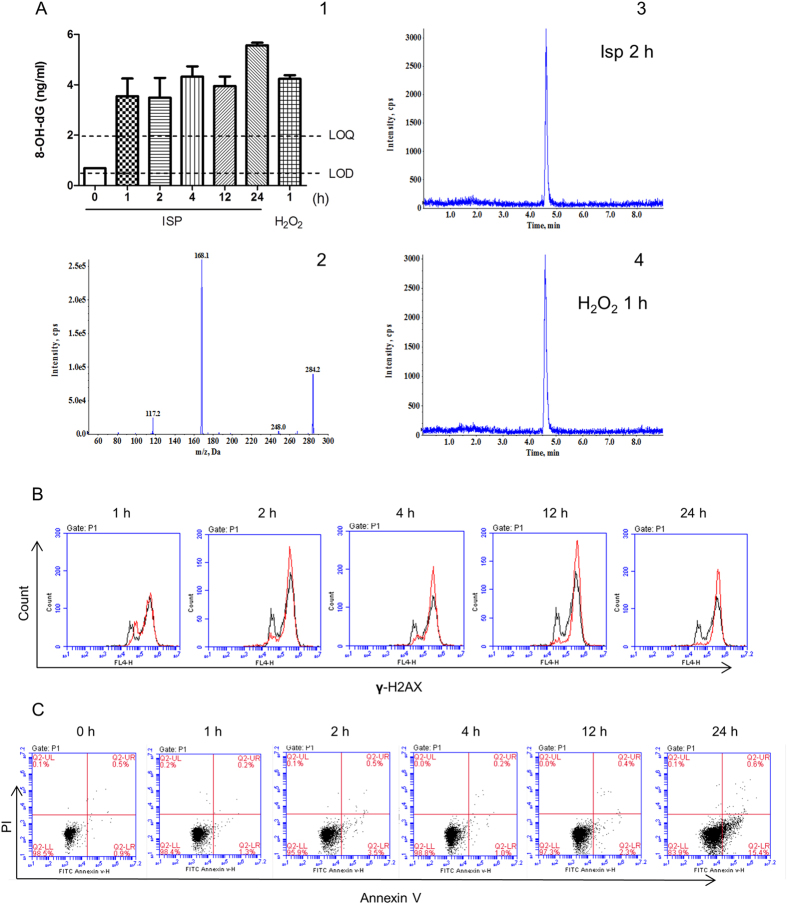
Time kinetic analysis of adrenergic stimulation-induced embryonic pluripotent cell DNA damage and apoptosis. (**A**) Increase of 8-OH-dG in ES cells induced by isoprenaline compared with the vehicle administrated control group assayed by LC-MS/MS (A1). Concentrations are expressed as means ± SEM of three independent experiments in triplicate samples. Where error bars are not shown, they lie within the dimensions of the symbol. The amount of 8-OH-dG in ES cells without treatment was below the limit of quantitation (LOQ), 2.0 ng/ML, but above the limit of detection (LOD), 0.5 ng/ML. Collision-induced dissociation (CID) mass spectra of 8-OH-dG are shown (A2). The most abundant and specific product ion was selected for multiple reaction monitoring (MRM) transition: m/z 284.2.0 [M + H] + → 168.1. Representative MRM chromatograms are shown for 8-OH-dG in the cells 2 h after isoprenaline treatment (A3) and 1 h after 100 μM H_2_O_2_ treatment (A4). The treatment with H_2_O_2_ was used as the positive control for the induction of 8-OH-dG. The method for the identification and quantitation of 8-OH-dG by LC-MS/MS is described in *Material and methods*. 8-OH-dG, 8-hydroxy-2′-deoxyguanosine. (**B**) Increase of γ-H2AX positive cells induced by isoprenaline in ES cells assayed by flow cytometry. A total of 10,000 cells was analyzed per sample. Black lines, control cells; red lines, isoprenaline treated cells. Isoprenaline was used at 10 μM for indicated duration. (**C**) Increase of Annexin V positive cells induced by isoprenaline in ES cells assayed by flow cytometry. A total of 10,000 cells was analyzed per sample. Isoprenaline was used at 10 μM for indicated duration.

Adrenergic mediators adrenaline and noradrenaline induced DNA damage in ES cells and ECS cells in a concentration-dependent manner, from 0.01 μM to 10 μM, as shown in western blotting analysis of γ-H2AX accumulation (Fig. 1A), one of the earliest and most sensitive markers of DNA damage for double-strand breaks^22^,^23^. The effect on the induction of DNA damage of adrenergic stress mediators was recapitulated by isoprenaline, an adrenergic β receptor agonist. Isoprenaline markedly induced γ-H2AX accumulation in ES cells assayed by western blotting (Fig. 1A), HCA (Fig. 1B), laser confocal imaging (Supplementary Fig. S1A), and flow cytometry (Fig. 1C). This effect was confirmed by comet assay for the analysis of DNA breaks and LC-MS-MS for the analysis of 8-hydroxy-2′-deoxyguanosine (8-OH-dG), a specific marker for oxidative DNA damage. Comet assay showed that isoprenaline and H_2_O_2_ induced the appearance of DNA breaks as the comet tails after single-cell gel electrophoresis (Fig. 1D). Moreover, the accumulation of 8-OH-dG in ES cells was markedly induced by isoprenaline stimulation assayed by LC-MS/MS (Fig. 2A and Supplementary Fig. S2).

Time kinetic experiment by LC-MS/MS showed that the accumulation of 8-OH-dG occurred at 1 h after the adrenergic stimulation and the increased levels of 8-OH-dG were maintained during the test duration of 24 h (Fig. 2A). This effect was confirmed by flow cytometry assay which showed that the damage occurred at 1 h after the adrenergic stimulation and the damage magnitude sustained during the test duration (Fig. 2B). Damage to DNA led to cell death. Flow cytometry assay on Annexin V/PI showed that ES cells (Fig. 2C) and ECS cells (Supplementary Fig. S3A) underwent apoptosis after the adrenergic stimulation, being more obvious at 24 h (Fig. 2C). Incubation of isoprenaline at 10 μM for 24 h increased the portion of Annexin V positive cells from 1.9 ± 0.85% to 16.2 ± 2.30%. The cell death were also confirmed by HCA of cell clone counting, which showed a decrease of ES cell clone formation number under the adrenergic stimulation (Supplementary Fig. S3B).

Neither adrenaline nor isoprenaline induced γ-H2AX accumulation in MEFs along the dose range (Supplementary Fig. S1B and C), indicating that embryonic pluripotent cells are more sensitive to the adrenergic stimulation-induced DNA damage.

During the time, adrenergic stimulation did not trigger the pluripotent stem cell differentiation as determined by monitoring the pluripotency markers Oct4 and Sox2 and the differentiation markers Nestin and Sox17 (Fig. 1B, Supplementary Fig. S3C, and data not shown).

### Adrenergic stimulation-induced DNA damage is caused by β2 adrenergic receptor signalling-generated intracellular ROS accumulation

**Figure 3:**
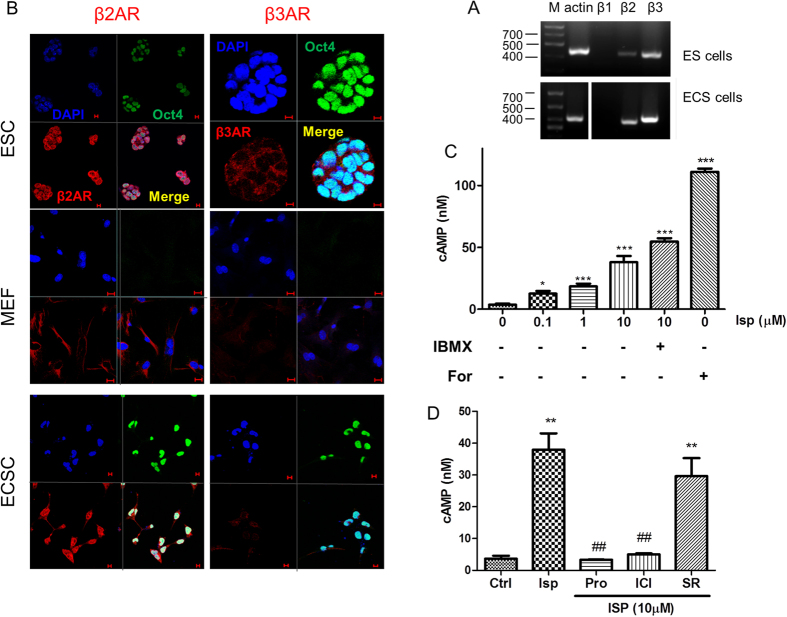
Functional expression of β2 adrenergic receptors in embryonic pluripotent cells. (**A**) RT-PCR analysis of β receptors in ES cells and ECS cells. (**B**) Confocal immunofluorescence staining analysis of β receptors in ES cells and ECS cells. DAPI represents cell nucleus position; Oct4 is a pluripotent marker. Scale bar: 20 μm. (**C**) β receptor activation-induced cAMP accumulation in ES cells. Forskolin, an adenylyl cyclase activator, and IBMX, a nonselective phosphodiesterase inhibitor that facilities cAMP accumulation behaved as the positive controls. Data represent means ± SEM from at least three independent experiments. *p < 0.05, ***p < 0.001, compared with the vehicle administrated control group. The cells were exposed to isoprenaline or forskolin for 30 min; IBMX was administrated 30 min before isoprenaline treatment. (**D**) β2 subtype receptor-dependent cAMP induction of adrenergic stimulation in ES cells. Pro, propranolol, a nonselective β receptor antagonist; ICI, ICI118551, a β2 receptor selective antagonist; SR, SR59230A, a β3 receptor selective antagonist. The cells were pretreated with 10 μM each of the antagonists for 30 min before the agonist treatment for 30 min. Data represent mean ± SEM from at least three independent experiments. **p < 0.01, compared with vehicle administrated control group; ^##^p < 0.01, compared with the isoprenaline administrated group. The gels have been run under the same experimental conditions. Full-length gels are presented in Supplementary Fig. S7.

The transcripts of β2 receptors and β3 receptors were expressed in ES cells and ECS cells as determined by RT-PCR analysis (Fig. 3A). Expression of the receptors was confirmed by immunocytofluorescence staining analysis in ES cells and ECS cells (Fig. 3B). β2 receptors or β3 receptors colocalized with the pluripotent marker Oct4 in the same undifferentiated pluripotent cells and the β2 receptors were expressed in higher intensity than the β3 receptors in the pluripotent cells (Fig. 3B). MEFs were also detected to express β2 receptors (Fig. 3B).

The β receptors in the embryonic pluripotent cells were functional because agonist stimulation of the receptors led to an accumulation of intracellular cAMP (Fig. 3C), the secondary messenger generated after the receptor activation via coupled Gs protein-adenylate cyclase (AC) signalling pathway. After pre-incubation with IBMX, a nonselective phosphodiesterase inhibitor, the β receptor activation-induced cAMP accumulation was augmented as expected and AC activator forskolin also markedly increase cAMP accumulation as expected (Fig. 3C).

The adrenergic activation-induced cAMP accumulation in embryonic pluripotent cells selectively depended on β2 receptors because the effect in ES cells was fully abrogated by nonselective β receptor antagonist propranolol or β2 receptor selective antagonist ICI118551 whereas SR59230A, a β3 receptor selective antagonist showed much less impact on the effect (Fig. 3D).

**Figure 4:**
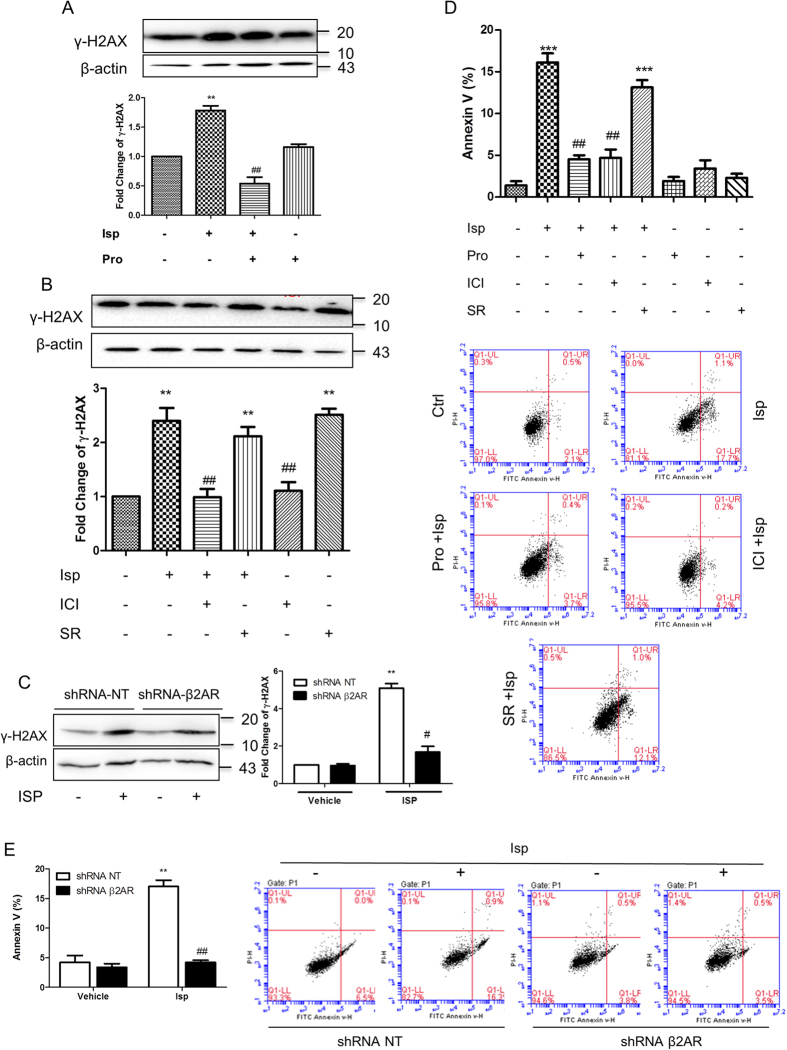
β2 subtype adrenergic receptor-mediated DNA damage and apoptosis of embryonic pluripotent cells. (**A,B**) β receptor-mediated (**A**) and β2 receptor-mediated (**B**) γ-H2AX accumulation in ES cells assayed by western blot. (**C**) Resistance to adrenergic stimulation-induced DNA damage in β2 receptor-knockdown ES cells. (**D**) β2 receptor-mediated ES cell apoptosis assayed by flow cytometry. (**E**) Resistance to adrenergic stimulation-induced apoptosis in β2 receptor-knockdown ES cells. Isoprenaline was used at 10 μM for 24 h. The antagonists each at 10 μM were administrated 45 min before the agonist treatment. Column graphs represent means ± SEM from three independent experiments. **p < 0.01, compared with the vehicle administrated control group; ^##^p < 0.01, compared with the isoprenaline administrated group. Adjacent to the column graphs, representative immunoblots or cytometry plots are shown. For HCA, quantification was averaged from at least six randomly selected microscopic fields. For flow cytometry analysis, a total of 10,000 cells was analyzed per sample. The gels have been run under the same experimental conditions. Full-length blots are presented in Supplementary Fig. S8 and S9.

Accordingly, the adrenergic stimulation-induced DNA damage in embryonic pluripotent cells also depended on β2 receptors. The DNA damage was blocked by propranolol, ICI118551, but not by SR59230A (Fig. 4A,B). Furthermore, when the β2 receptors of the ES cells were knocked down, the cells showed fully resistant to isoprenaline-induced DNA damage (Fig. 4C). This also indicates that the DNA damage-induction effect is directly ascribed to the β2 receptors expressed in ES cells rather than indirectly to those of MEFs.

Damage to DNA leads to ES cell apoptosis. Adrenergic stimulation markedly increased the portion of Annexin V-positive ES cells. The effect was abrogated by β receptor blockade or β2 receptor-specific blockade whereas β3 receptor-specific blockade failed to inhibit the effect (Fig. 4D). The β2 receptor-mediated mechanism was confirmed by the knockdown of β2 receptors in ES cells. In these cells, the adrenergic stimulation-induced apoptosis was completely abrogated (Fig. 4E).

**Figure 5:**
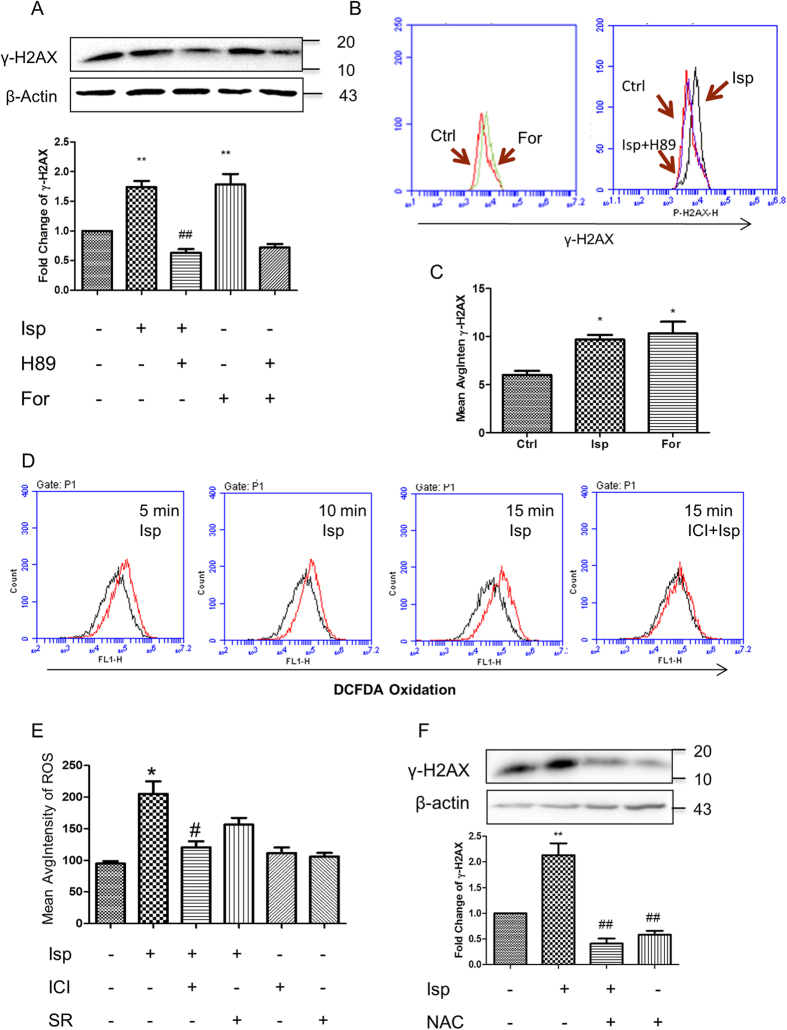
AC/cAMP/PKA signalling and ROS dependence of adrenergic stimulation-induced embryonic pluripotent cell DNA damage. (**A**) Effect of AC/cAMP/PKA signalling on γ-H2AX accumulation in ES cells assayed by western blot. AC activator forskolin increased the accumulation of γ-H2AX in ES cells. PKA inhibitor H89 abrogated forskolin- and adrenergic stimulation-induced DNA damage. (**B,C**) Effect of AC/cAMP/PKA signalling on γ-H2AX accumulation in ES cells assayed by flow cytometry (**B**) and HCA (**C**). (**D,E**) β2 receptor activation-generated accumulation of intracellular ROS assayed by flow cytometry (**D**) and HCA (**E**). (**F**) Abrogation of adrenergic activation-induced γ-H2AX accumulation by ROS quenching. NAC, N-acetyl-L-cysteine. Representative immunoblots are shown above the column graphs (**A,F**). Isoprenaline was used at 10 μM for 15 min (**E**), 24 h (**A–C,F**), or indicated duration (**D**). Forskolin was used at 10 μM for 24 h. H89 at 20 μM was administrated 45 min before the agonist or forskolin treatment. The antagonists ICI or SR at 10 μM or the ROS quencher NAC at 3 μM were administrated 30 min before the agonist treatment. Column data represent means ± SEM from three independent experiments. *p < 0.05, **p < 0.01, compared with the vehicle administrated control group; ^#^p < 0.05, ^##^p < 0.01, compared with the isoprenaline administrated group. For HCA, quantification was averaged from at least six randomly selected microscopic fields. The gels have been run under the same experimental conditions. Full-length blots are presented in Supplementary Fig. S10 and S11.

Stimulation of β2 receptor initiates the activation of AC, leading to an increase of cAMP and subsequent activation of PKA. Accordingly, AC activator forskolin increased an accumulation of γ-H2AX in ES cells, mimicking the effect of β2 receptor activation whereas PKA inhibitor H89 markedly abrogated forskolin- and adrenergic stimulation-induced DNA damage assayed by Western blot (Fig. 5A), flow cytometry (Fig. 5B) and HCA (Fig. 5C).

In addition to AC/cAMP/PKA signalling pathway, β-arrestin/MDM2/p53 pathway has been reported to partially contribute to β receptor stimulation-induced DNA damage in somatic cells^11^. In ES cells, isoprenaline in 10 μM did not induce MDM2 activation or downregulate p53 expression (Supplementary Fig. S4A–C). Moreover, the knockdown of Arrb1 (β-arrestin-1) or Arrb2 (β-arrestin-2) in ES cells failed to abrogate the β receptor stimulation-induced DNA damage (Supplementary Fig. S4D,E). These indicate that β receptor stimulation-induced DNA damage is mainly mediated by the receptor coupled AC/cAMP/PKA signalling pathway in the embryonic pluripotent cells.

Generation of intracellular ROS is one of the prominent causes leading to DNA damage. Stimulation of β receptors increased intracellular ROS content in ES cells as showed by flow cytometry (Fig. 5D) and HCA (Fig. 5E) using redox sensitive fluorescent probe DCFDA. The increased levels of ROS were maintained during the time course of test, from 5 min after exposure to isoprenaline until 15 min (Fig. 5D). This indicates an adrenergic stimulation-induced source of ROS accumulation. Accordingly the amount of oxidative DNA damage marker 8-OH-dG was considerably increased (Supplementary Fig. S5). The effect was significantly abrogated by β2 receptor blockade (Fig. 5D,E and Supplementary Fig. S5). When the accumulated ROS was quenched by NAC, the isoprenaline-induced γ-H2AX accumulation was blocked (Fig. 5F), suggesting the role of ROS on the the adrenergic stimulation-caused DNA damage.

## Discussion

The key finding in this study is that adrenergic stress mediators causes DNA damage of embryonic pluripotent cells through the induction of intracellular ROS accumulation and that the effect is mediated by β2 adrenergic receptor and its cognate AC/cAMP/PKA signalling pathway.

Genotoxic effects of radiation and xenobiotics on embryonic pluripotent cells have been extensively studied whereas those from stress mediators are much less explored. Discovering the genotoxic properties of adrenergic stress mediators to embryonic pluripotent cells and delineating the underlying receptor subtype mechanism will improve the understanding of stem cell biology and physiology/pathophysiology and help to design strategies to modulate/protect the stem cells by targeting adrenergic signalling pathways in a selective way.

Adrenergic responses prepare the body for coping with the perceived threats and are crucial for the adaptation and harmony of the organism^3^,^24^. However, excessive adrenergic stress responses involving the stress mediator-induced cell DNA damage in somatic cells impair body homeostasis and contribute to numerous diseases^5^,^9^,^10^,^11^,^12^,^25^,^26^,^27^. In hematopoietic progenitor cells, adrenergic agonists have been reported to induce DNA damage and inhibition of cell proliferation^28^. In embryonic pluripotent cells, we for the first time found that the adrenergic stress agonists adrenaline, noradrenaline, and isoprenaline caused DNA damage and apoptosis. The effects were mimicked by β2 receptor-coupled signalling molecules and abrogated by selective blockade of β2 receptors and inhibition of the receptor signalling pathway. RNA interference targeting β2 receptors of ES cells conferred the cells the ability to resist the adrenergic stress-induced DNA damage and apoptosis. In addition, adrenergic stimulation caused a consistent accumulation of ROS in ES cells, and the effect was abrogated by β2 receptor blockade. Quenching of ROS reversed the adrenergic stress-induced DNA damage, indicating the involvement of ROS accumulation of β2 receptor signalling for the induction of the damage.

ROS can be endogenously produced during oxidative metabolism. Though important for physiological processes such as signal transduction, excessive generation of ROS causes damage to DNA in somatic and stem cells^29^,^30^. Keeping a balanced ROS level is crucial for the homeostasis and well-being of the organism. In hematopoietic stem cells, ROS accumulation and ROS-induced DNA damage limits the lifespan of the cells and contributes to the exhaustion of the stem cell population and the dysfunction of the cells^30^,^31^. Compared with somatic cells, stem cells bear a lower baseline level of oxidative burden to protect genome integration by low mitochondrial biogenesis, reduced oxygen consumption and ATP generation through anaerobic glycolysis rather than oxidative phosphorylation and are sensitive to increased ROS^32^,^33^. Adrenergic stress induced an marked accumulation of ROS in embryonic pluripotent cells, thus causing a damage to DNA.

Activation of β2 receptors increased ROS in embryonic pluripotent cells via AC/cAMP-PKA signalling pathway. In cardiomyocytes, β2 receptor stimulation induces ROS accumulation via the activation of NADPH oxidase, leading to cardiomyopathy^34^. Activation of cAMP/PKA signalling similarly results in an increase in mitochondrial ROS production^35^,^36^. The increased ROS production induced by β receptor stimulation is a direct consequence of cAMP–PKA-dependent signalling and relies on the modulation of SOD expression or mitochondria function by the receptor signalling^35^,^37^,^38^,^39^.

Damage to DNA triggers a cascade of signalling events defined as the DNA damage response (DDR) including DNA repair^1^,^40^. After DNA damage induced by adrenergic stimulation, embryonic pluripotent cells underwent apoptosis, suggesting that the damage was too excessive to be properly corrected and that the cells were then programmed to undergo apoptosis to eliminate potentially dangerous cells carrying defective DNA. This will limit the transmission of the damaged genome to the progeny cells^41^. While this mechanism prevent mutations from propagating to the developing embryos, it depletes stem cell pool and potentially impairs tissue maintenance and induces aging^42^. The embryonic pluripotent cells bearing damaged DNA underwent apoptosis and maintained their pluripotency markers, consistent with the report that ES cells are able to keep their self-renewal capacity under the H_2_O_2_-induced cell cycle arrest and apoptosis^43^. This implies that stem cells are able to take advantage of cell cycle checkpoints trying to limit genome lesion and maintain self-renewal^44^. Cell cycle arrest, allowing time and opportunity for the repair of DNA, is one of the core events in DDR^1^. We have found that under adrenergic stress mediator stimulation, embryonic pluripotent cells undergo cell cycle arrest and proliferation inhibition^45^. It would be worth further exploring whether the cell cycle arrest is attributed to the induced DNA damage response.

DNA damage, which is apt to leads to mutation and genome instability, must be keep low in ES cells and early embryos in this critical stage of development^46^,^47^. Defects in their genome will have devastating consequences and even affect subsequent generations when the cells recreate the entire organism and the germ cells. Accumulated DNA damage is also a principal mechanism underlying stem decline in many diseases^48^. Thus adrenergic stress, which causes DNA damage via induction of the accumulation of ROS by activation of β2 receptor signalling, will compromise embryonic pluripotent cells and the well-being of the whole organism. It is worth confirming whether β receptors represent a novel target for the protection of embryonic pluripotent cells from DNA damage and subsequent genome instability insulted by adrenergic stress. It is expecting in terms of a translational view considering the great success of β receptor blockers in drug development and disease therapy^49^,^50^.

## Material and Methods

### Materials

Dulbecco’s Modified Eagle’s Medium (DMEM), KnockOut-Dulbecco’s modified eagle medium (KO-DMEM), fetal bovine serum (FBS), KnockOut Serum Replacement (KSR), β-mercaptoethanol, L-glutamine, non-essential amino acids (NEAA), and GlutaMAX were purchased from GIBCO/Life Science. Leukemia inhibitory factor (LIF) was purchased from Chemicon. Isoprenaline, ICI118551, and SR59230A were purchased from Tocris Bioscience. 5-(and-6)-carboxy-2′,7′-dichlorofluorescin diacetate (carboxy-DCFDA), 8-Hydroxy-2′-deoxyguanosine (8-OH-dG) and N-acetyl-L-cysteine (NAC) were purchased from Sigma-Aldrich. Forskolin and H89 were purchased from Beyotime. HPLC-grade methanol, water, and formic acid were purchased from Merck. Rabbit polyclonal anti-β3 receptor antibody, mouse monoclonal anti-Oct4 antibody, rabbit polyclonal anti-MDM2 antibody, and mouse monoclonal anti-β-Arrestin-1/2 antibody were purchased from Santa Cruz. Rabbit polyclonal anti-β2 receptor antibody was purchased from Abcam. Rabbit monoclonal anti-phospho-histone H2AX antibody, mouse monoclonal anti-p53 antibody, and rabbit polyclonal anti-phospho-MDM2 (Ser166) antibody were purchased from Cell Signalling Technology.

### Cell culture

The mouse R1 ES cell line was obtained from the Institute of Biochemistry and Cell Biology of the Chinese Academy of Sciences (Shanghai, China). The mouse P19 ECS cell line was obtained from the Cell Bank of Type Culture Collection of the Chinese Academy of Sciences (Shanghai, China). The culture and monitoring of pluripotency of the embryonic pluripotent cells were performed as described in our previous report^51^,^52^. ES cells were grown with irradiated mouse embryonic fibroblasts (MEFs) as feeder layer in KO-DMEM supplemented with 1/10000 (v/v) LIF, 1/500 (v/v) β-mercaptoethanol, 1/100 (v/v) L-glutamine, 1/100 (v/v) non-essential amino acids, and 15% FBS. ECS cells were grown in DMEM supplemented with 10% FBS and 1% GlutaMAX. The cells were maintained at 37 °C in a humidified atmosphere containing 5% CO_2_ and 95% air.

### Western blotting

Cell lysates were resolved by 10% sodium dodecyl sulfate–polyacrylamide gel electrophoresis (SDS-PAGE), transferred onto PVDF membranes (Millipore), blocked in 5% nonfat milk, and incubated with appropriate primary antibodies overnight at 4 °C. After incubated with the corresponding secondary antibodies the immunoblots were visualized and scanned by using the Odyssey FC Imaging System (LI-COR Biosciences).

### High-content analysis

Image-based high-content analysis^53^,^54^ was performed by using the HCA System (ArrayScan XTI, Thermo Scientific). Cells were incubated with appropriate first antibodies and then the corresponding fluorescence-conjugated second antibodies for the quantitative determination of the protein expression levels. Cell nuclei were counterstained with DAPI and the cell pluripotency was monitored by Oct4 immunofluorescence staining.

### Comet assay (Single cell gel electrophoresis assay)

The alkaline comet assay was carried out as previously described with minor modifications^55^. The cells were mixed with 0.5% low-melting agarose and applied to glass slides pre-coated with 1% normal-melting agarose. Slides mounted with cells were immersed in cold lysing solution (2.5 M NaCl, 100 mM EDTA, 10 mM Tris base, 1% Triton X-100, 10% DMSO, 200 mM NaOH, pH = 10) for 2.5 h at 4 °C. Then electrophoresis was conducted at 300 mA for 20 min in the dark. After neutralized in 0.4 M Tris-HCl for 5 min, the slides were dipped in ethanol and air-dried in the dark. Cells were stained with SYBR Green I and observed by using a fluorescence microscope (Leica TCS-NT). A total of 100 cells per sample was analyzed for the percentage of DNA in the comet tail by using the Komet software program v5.5.

### Flow cytometry

Cell samples were analyzed with a flow cytometer (Accuri C6, BD Biosciences). Fixed and permeated cells were incubated with anti-phospho-histone H2AX antibody and the corresponding fluorescence-conjugated second antibody for the detection of DNA damage. The fluorescent oxidation indicator carboxy-DCFDA was used to quantify the intracellular reactive oxygen species (ROS) levels. Annexin V/PI apoptosis detection Kit (BD Biosciences) was used to determine cell apoptosis. A total of 10,000 cells was analyzed per sample.

### Measurement of 8-OH-dG content by Liquid chromatography/mass spectrometry (LC–MS/MS)

Calibration standards were prepared at concentrations of 2, 5, 10, 20, 50 and 100 ng/mL by diluting a fixed amount of stock solution of 8-OH-dG in methanol/water (4:1, v/v) in tubes. All calibration standards were stored at −70 °C until the LC-MS/MS analysis.

LC was performed on a Shimadzu prominence UFLC-XR system (Shimadzu), and separation was carried out at 35 °C by using an Agilent Eclipse XDB-C18 column (4.6 × 150 mm, 5 μm). Methanol (solvent A) and 0.1% formic acid in water (solvent B) were used as mobile phases, and the flow rate was set at 0.30 mL/min. The gradient started with 60% of mobile phase A, increasing to 100% at 6 min. The mobile phase was held isocratic for 1 min before returning to 60% mobile phase A, then for 2 min to equilibrate the column and reestablish the C18 condition (total run time was 9 min). The injection volume was 5 μL.

The LC system was coupled to a triple quadrupole mass spectrometer (API-4000, AB SCIEX). Conditions for Declustering Potential (DP), Collision Energy (CE) and Exit Cell Potential (CXP) for each analyte were obtained by using the Quantitative Optimization function in the analyst software. An electrospray ionization (ESI) in positive ionization mode was used. Unless otherwise specified, the ESI source parameters were set as: curtain gas, 35 psi; gas flows (GS1, GS2), 50 psi; gas temperature (TEM), 550 °C. The mode of multiple reaction monitoring (MRM) was used to identify and quantify 8-OH-dG (transition: m/z 284.2 [M + H] + → 168.1, DP 50 V, collision energy 15 eV).

In sample preparation procedure, 100 μL of sample was mixed with 200 μL of chilled methanol (containing 0.3% formic acid). The mixture was vortexed for 30 seconds and centrifuged at 13,000 g, for 10 min. The supernatant (5 μL) was subjected to the LC-MS/MS analysis.

Other LC-MS/MS procedures were performed as described in our previous reports^52^,^56^,^57^.

### RNA isolation and reverse transcript PCR

Total RNA from cells was isolated with RNA Extraction Kit (Omega) according to the manufacturer’s instructions. Reverse transcription (RT) was carried out using a RevertAid First Strand cDNA Synthesis Kit (Invitrogen). The resultant cDNA was amplified according to the following temperature profile: 94 °C for 30 s, 55 °C for 45 s, and 72 °C for 1 min. At the end of 31 cycles, the reaction was continued for an additional 10 min at 72 °C. The amplified cDNA was subjected to 2% agarose gel electrophoresis. The primer sequences used for reverse transcript PCR are shown in Supplementary Table S1:

### Immunofluorescence staining

Cells were seeded into multiple glass-bottom tissue culture plates (10 mm; Shengyou Biotechnology) and cultured for 24 h. After fixed the cells were blocked with 1% bovine serum albumin (BSA) for 60 min at 37 °C and incubated overnight at 4 °C with primary antibodies (1:400 for anti-phospho-H2AX, 1:300 for anti-β2 receptor, 1:300 for anti-β3 receptor, and 1:200 for anti-Oct4). Next, the cells were incubated for 1 h at 37 °C with the corresponding secondary antibodies and then subjected to laser confocal microscopy (Leica SP8) analysis.

### RNA interference

β2-AR shRNA and nontarget control shRNA lentiviral particles were purchased from Santa Cruz Biotechnology. The RNA interference procedure was performed according to the manufacturer’s instruction. Levels of the receptor protein expression were detected by western blotting.

### Determination of cAMP level

Cells were detached and resuspended in assay buffer. Intracellular cAMP determination assay was carried out by using a cAMP dynamic kit based on homogenous time-resolved fluorescence (HTRF) technology according to the manufacturer’s instructions (CisBio International) and the florescent signals were read in a plate reader (EnVision, Perkin–Elmer).

### Statistical analysis

Statistical significance was tested by using a Student’s test or an one-way ANOVA with Bonferroni post-test properly. Differences were considered statistically significant when P < 0.05.

## Additional Information

**How to cite this article**: Sun, F. *et al*. Adrenergic DNA damage of embryonic pluripotent cells via β2 receptor signalling. *Sci. Rep*. **5**, 15950; doi: 10.1038/srep15950 (2015).

## Supplementary Material

Supplementary Information
