# Supplementary material for: Adrenergic DNA damage of embryonic pluripotent cells via β2 receptor signalling
Source: Sci Rep. 2015 Oct 30;5:15950. doi: 10.1038/srep15950 (PMC4626766; doi:10.1038/srep15950)
Supplement: Supplementary Information [file srep15950-s1.doc]

**SUPPLEMENTARY INFORMATION**

**Adrenergic DNA damage of embryonic pluripotent cells via β2 receptor signalling**

Fan Sun ^1,3,#^, Xu-Ping Ding ^1,#^, Shi-Min An ^1,2^, Ya-Bin Tang ^1,2^, Xin-Jie Yang ^1^, Lin Teng ^1,5^, Chun Zhang ^1,4^, Ying Shen ^1,2^, Hong-Zhuan Chen ^1,2, *^ , Liang Zhu ^1,2, *^


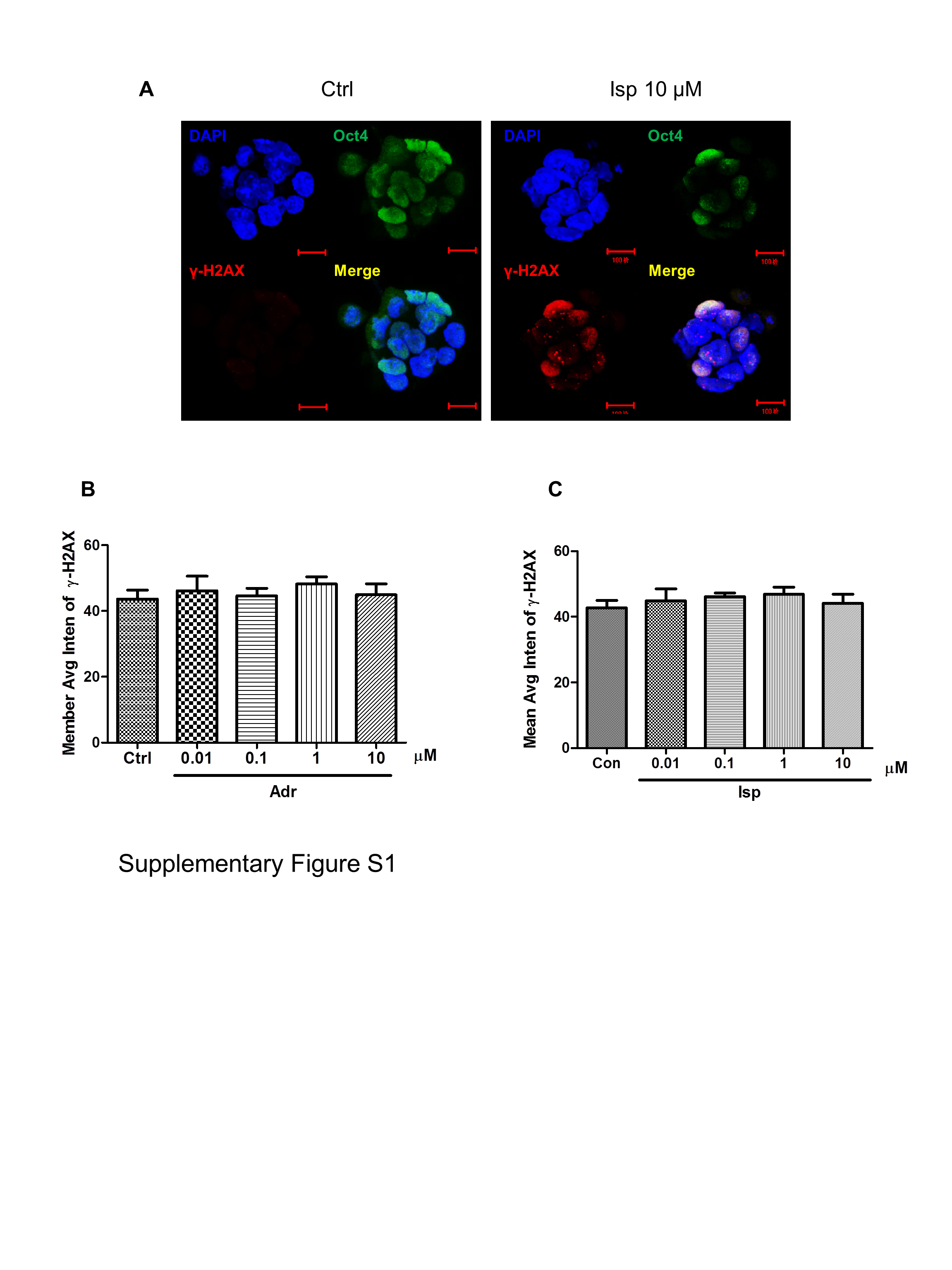


Supplementary Figure S1. Induction of increase of γ-H2AX foci formation in embryonic stem cell nuclei and non-induction of DNA damage on mouse embryonic fibroblasts by adrenergic stimulation.

(A) Induction of γ-H2AX foci accumulation by 10 μM isoprenaline for 24 h in ES cells imaged by laser confocal microscopy. Scale bar: 50 μm.

(B and C) Effect of adrenaline (B) and isoprenaline (C) treatment on γ-H2AX accumulation in MEFs analyzed by HCA. The cells were treated by the adrenergic agonists at the indicated concentrations for 24 h. Quantification was averaged from at least six randomly selected microscopic fields. Data represent means ± SEM from at least three independent experiments.


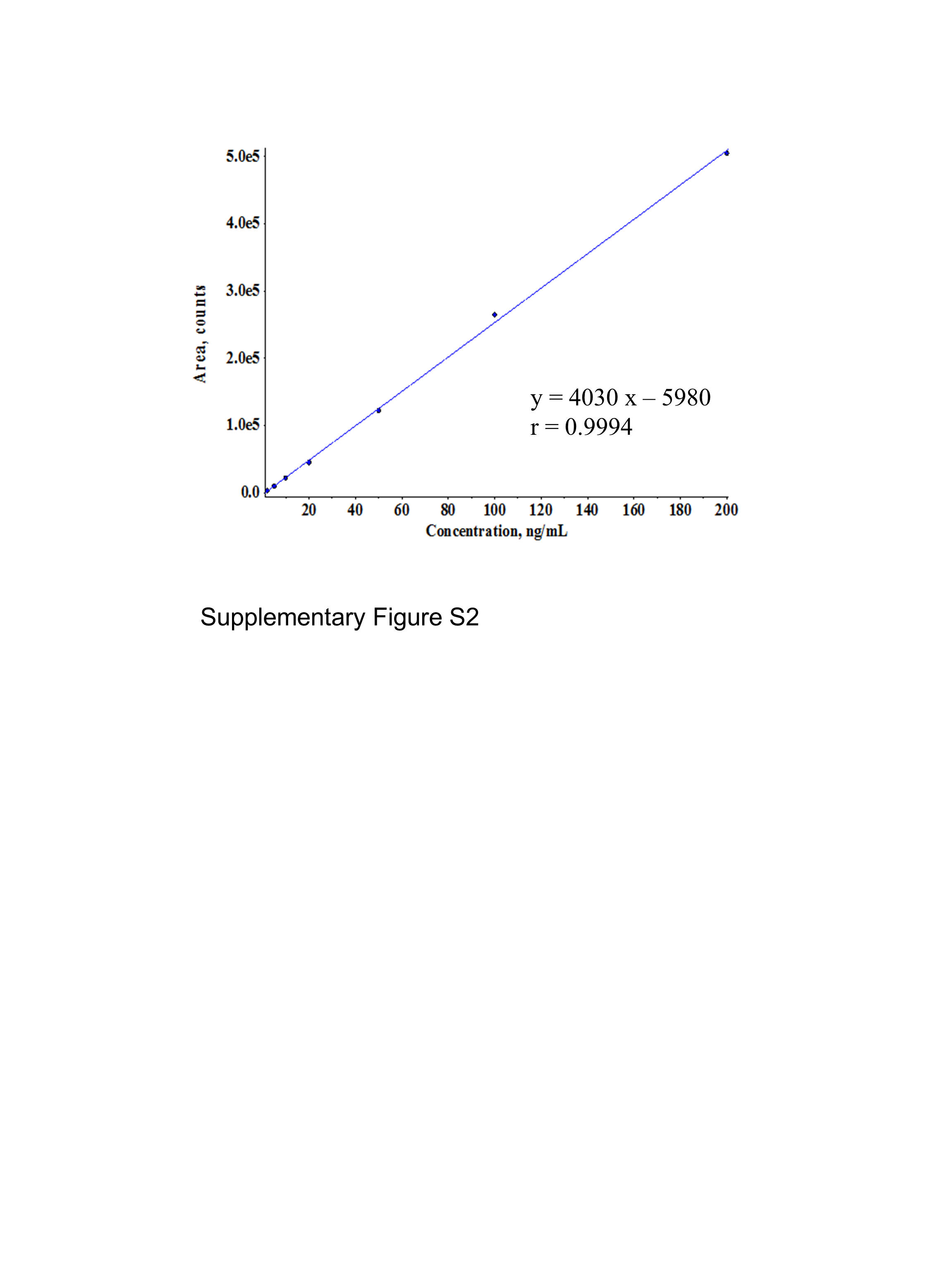


Supplementary Figure S2. The calibration curve for 8-hydroxy-2'-deoxyguanosine.

Typical daily calibration curve for 8-hydroxy-2'-deoxyguanosine showing a linear relationship between the peak area of the analyte (y) and analyte level (x).


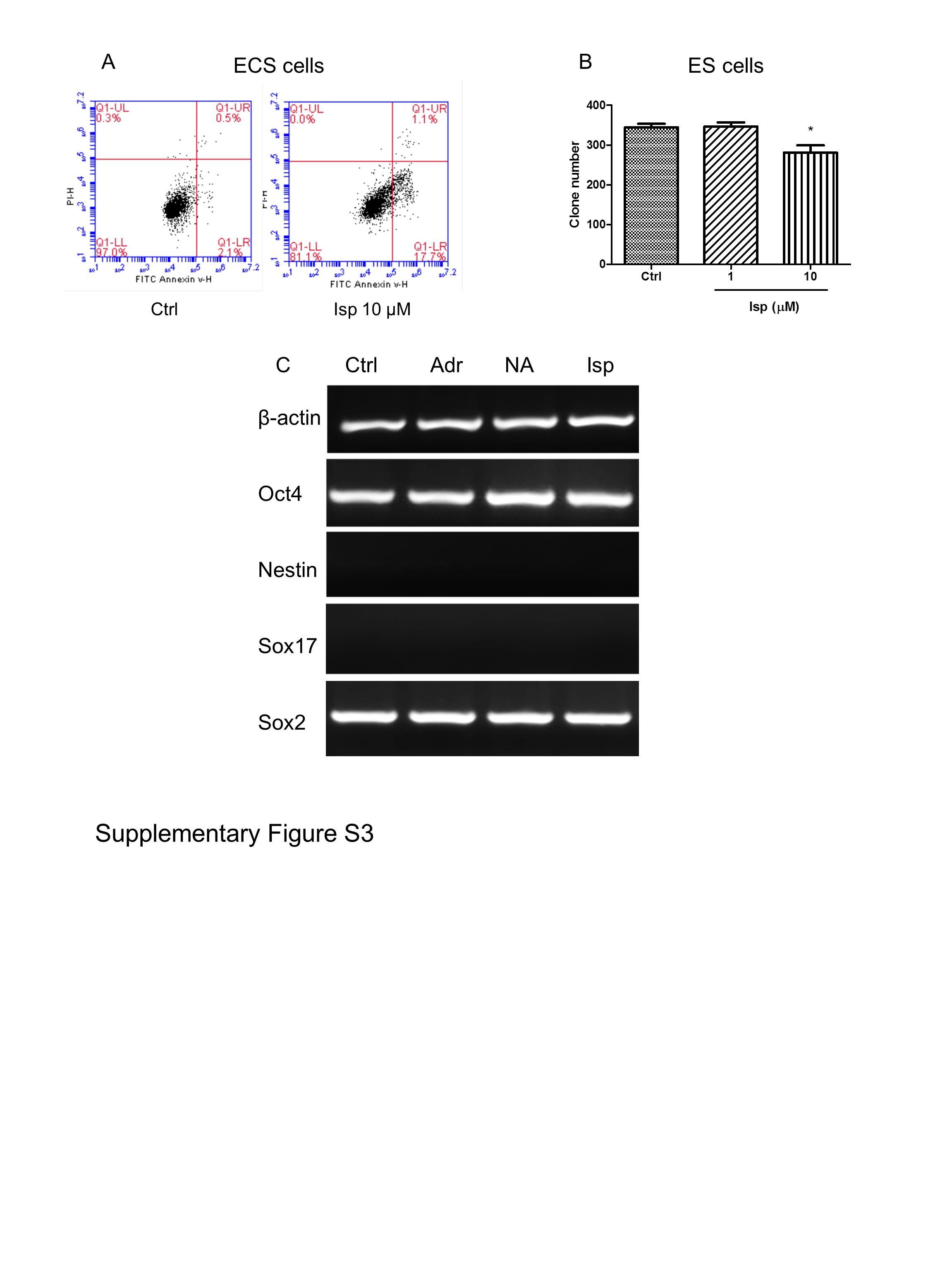


Supplementary Figure S3. The effect on the cell death and levels of the pluripotency and the differentiation markers under the adrenergic stimulation on embryonic stem cells.

(A and B) Induction of apoptosis of embryonal carcinoma stem cells (A) and decrease of survival of embryonic stem cells (B) by adrenergic stimulation assayed by flow cytometry and HCA, respectively. The cells were treated by isoprenaline at indicated concentration for 24 h.

(C) Levels of the pluripotency and the differentiation markers under the adrenergic stimulation on embryonic stem cells. The ES cells were treated by the adrenergic agonists adrenaline, noradrenaline, and isoprenaline each at 10 μM for 24 h. Oct4 and Sox2 are pluripotency markers; Nestin and Sox17 are differentiation markers. Their expression was assayed by RT-PCR.


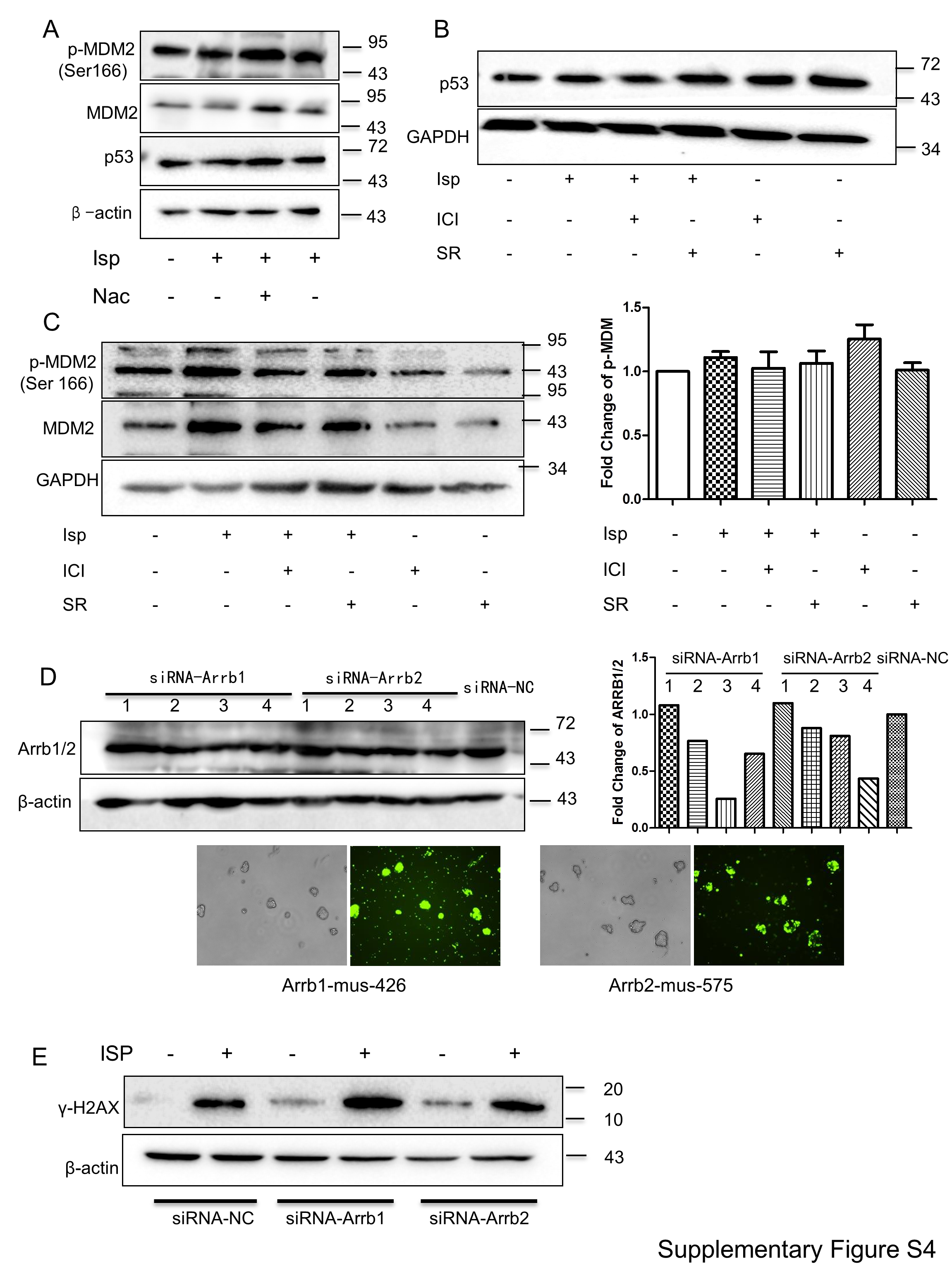


Supplementary Figure S4. Effects of β-arrestin/MDM2/p53 signalling pathway on the β receptor stimulation-induced DNA damage.

(A) Effect of isoprenaline and N-acetyl-L-cysteine on the expression level of MDM2 phosphorylation and p53. Representative blots in two independent experiments are shown.

(B) Effect of isoprenaline and the adrenergic antagonists on the level of p53 expression. Representative blots in three independent experiments are shown.

(C) Effect of isoprenaline and the adrenergic antagonists on the MDM2 phosphorylation. Data in the column graphs show the densitometric analysis.

(D) Knockdown of β-arrestin1/2 by siRNA. Western blot shows the expression of β-arrestin-1 (Arrb1) and β-arrestin-2 (Arrb2) 24 h after the siRNA-Arrb1 and siRNA-Arrb2 were transfected into the cells. Column graphs show the densitometric analysis relative to β-action control expressed as the fold change compared to shRNA-NC group. The cells transfected with the 3# siRNA-Arrb1 (Arrb1-mus-426) and the 4# siRNA-Arrb2 (Arrb2-mus-575) were chosen to detect the response under the adrenergic stimulation treatment. Immunofluorescence image (20 x magnification objective lens) shows the FAM-siRNAs transfected into the cells.

(E) Knockdown of β-arrestin1/2 by siRNA did not abrogate the isoprenaline-induced DNA damage.

Isoprenaline was used at 10 μM for 24 h. The antagonists each at 10 μM or NAC at 3 μM were administrated 30 min before the agonist treatment.


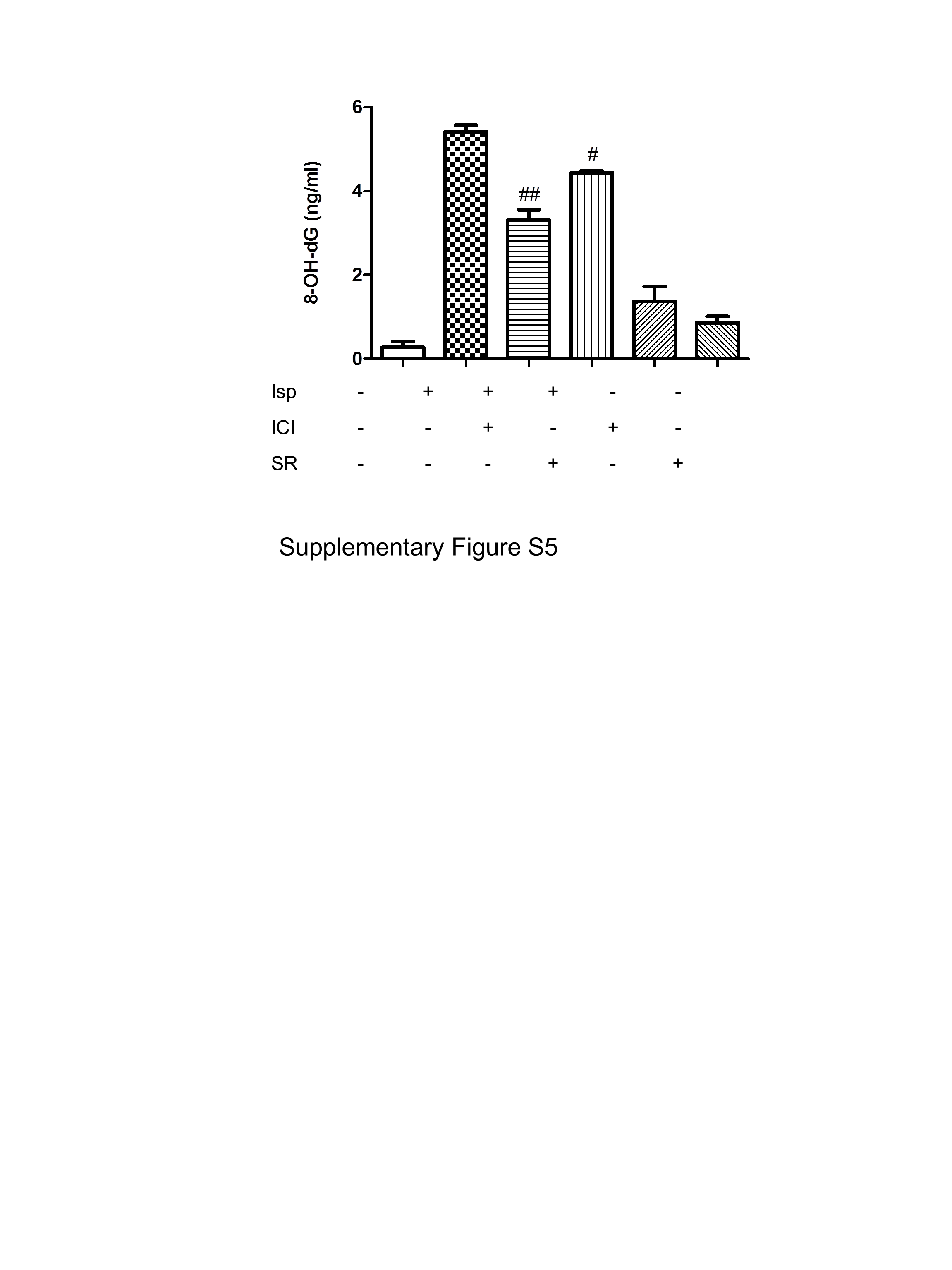


Supplementary Figure S5. β2 subtype adrenergic receptor-mediated increase of oxidative marker for DNA damage.

LC-/MS/MS was used to identify and quantify 8-OH-dG (see *Material and methods* for details). The antagonists ICI or SR at 10 μM were administrated 30 min before the agonist treatment. Concentrations are expressed as means ± SEM of three independent experiments in triplicate samples. Where error bars are not shown, they lie within the dimensions of the symbol. LOQ, limit of quantitation. #, p < 0.05, ##, p < 0.01, compared with the isoprenaline administrated group.


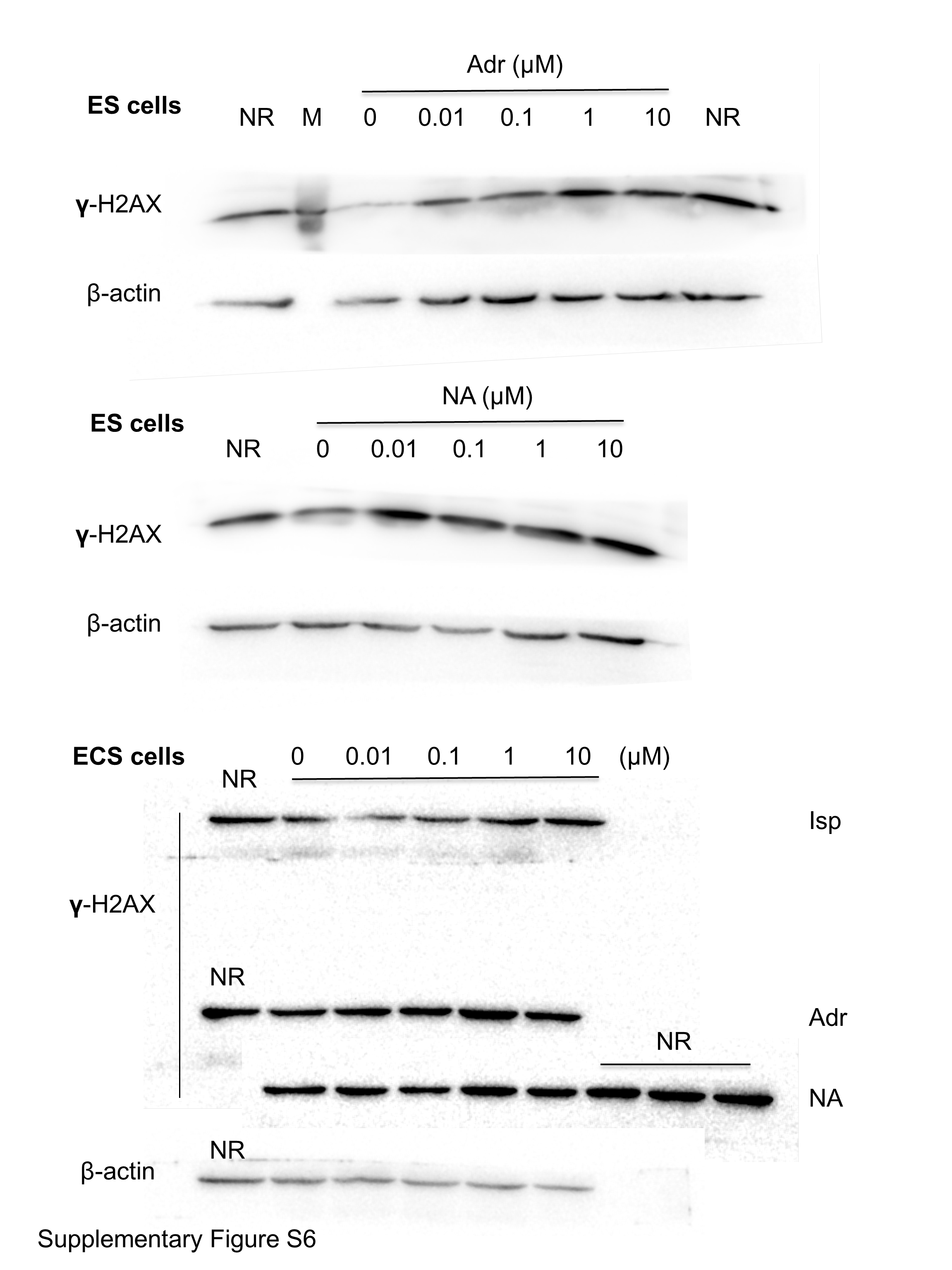


Supplementary Figure S6. Immunoblot analysis of γ-H2AX accumulation in ES cells and ECS cells induced by adrenergic agonists. Adr, adrenaline; NA, noradrenaline; Isp, isoprenaline. M, marker; NR, non-relevant lane-occupancy loading.


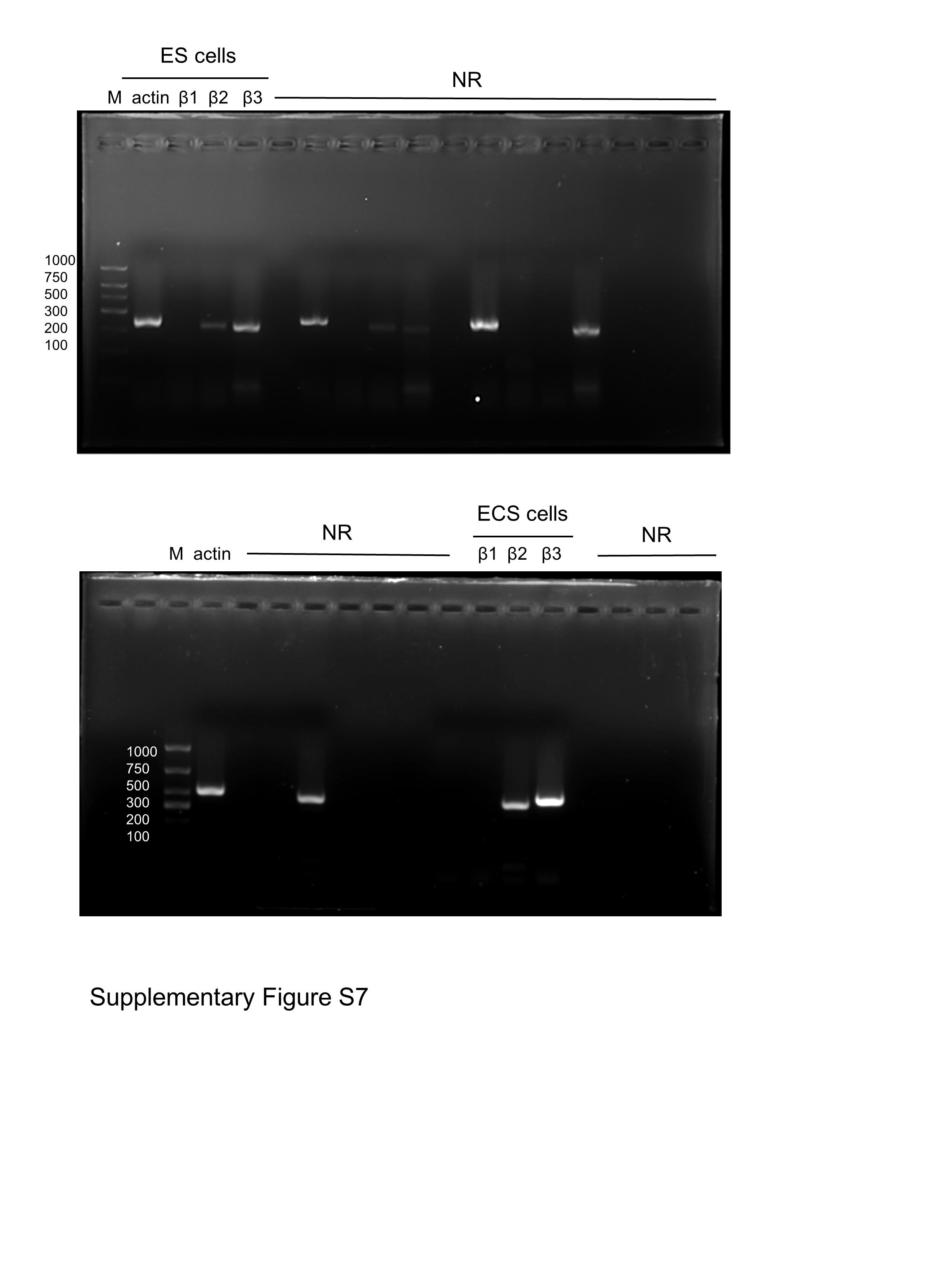


Supplementary Figure S7. RT-PCR analysis of β receptors in ES cells and ECS cells. M, marker; NR, non-relevant loading for other purpose.


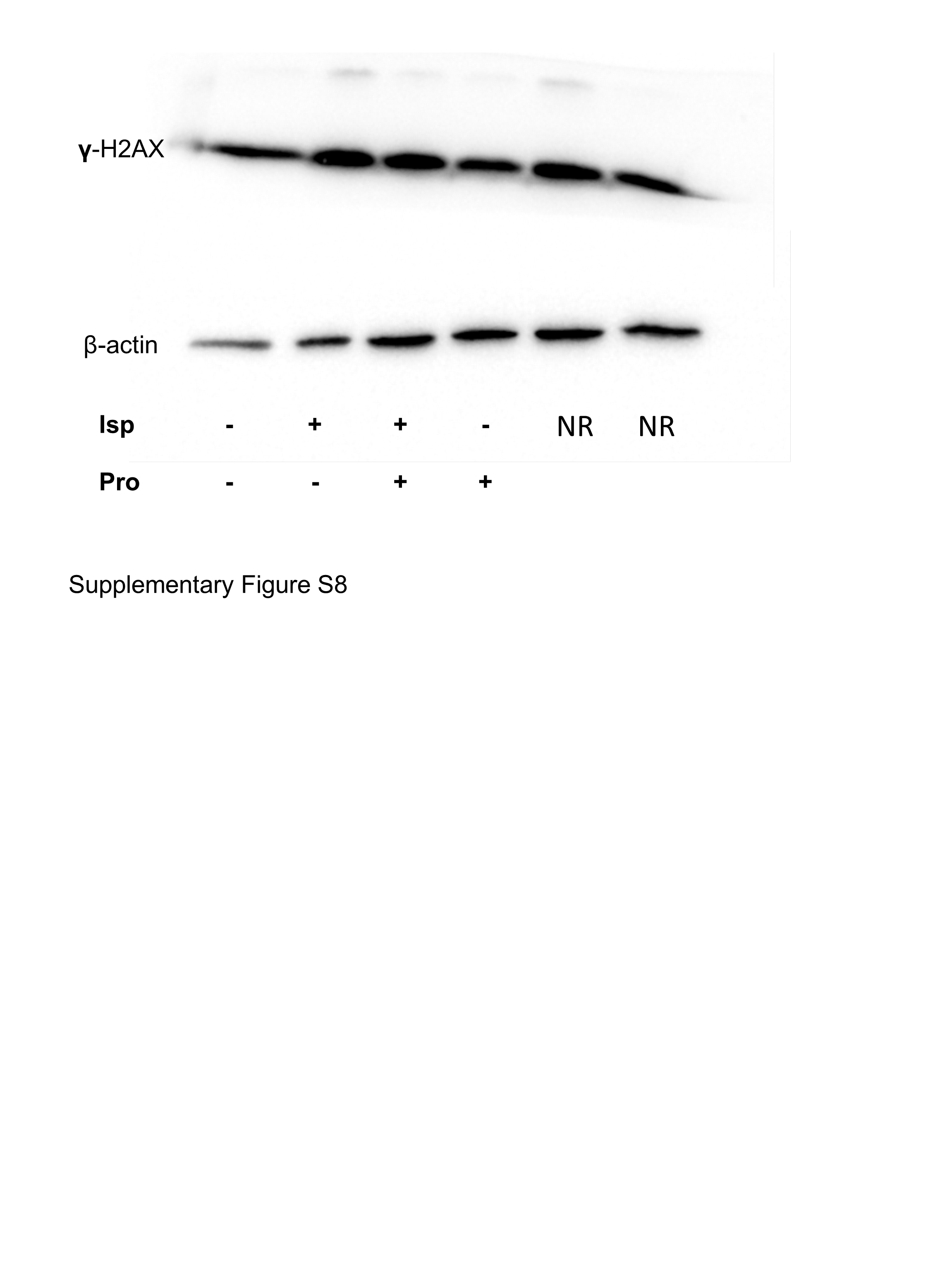


Supplementary Figure S8. Immunoblot analysis of β receptor-mediated γ-H2AX accumulation in ES cells. NR, non-relevant lane-occupancy loading.


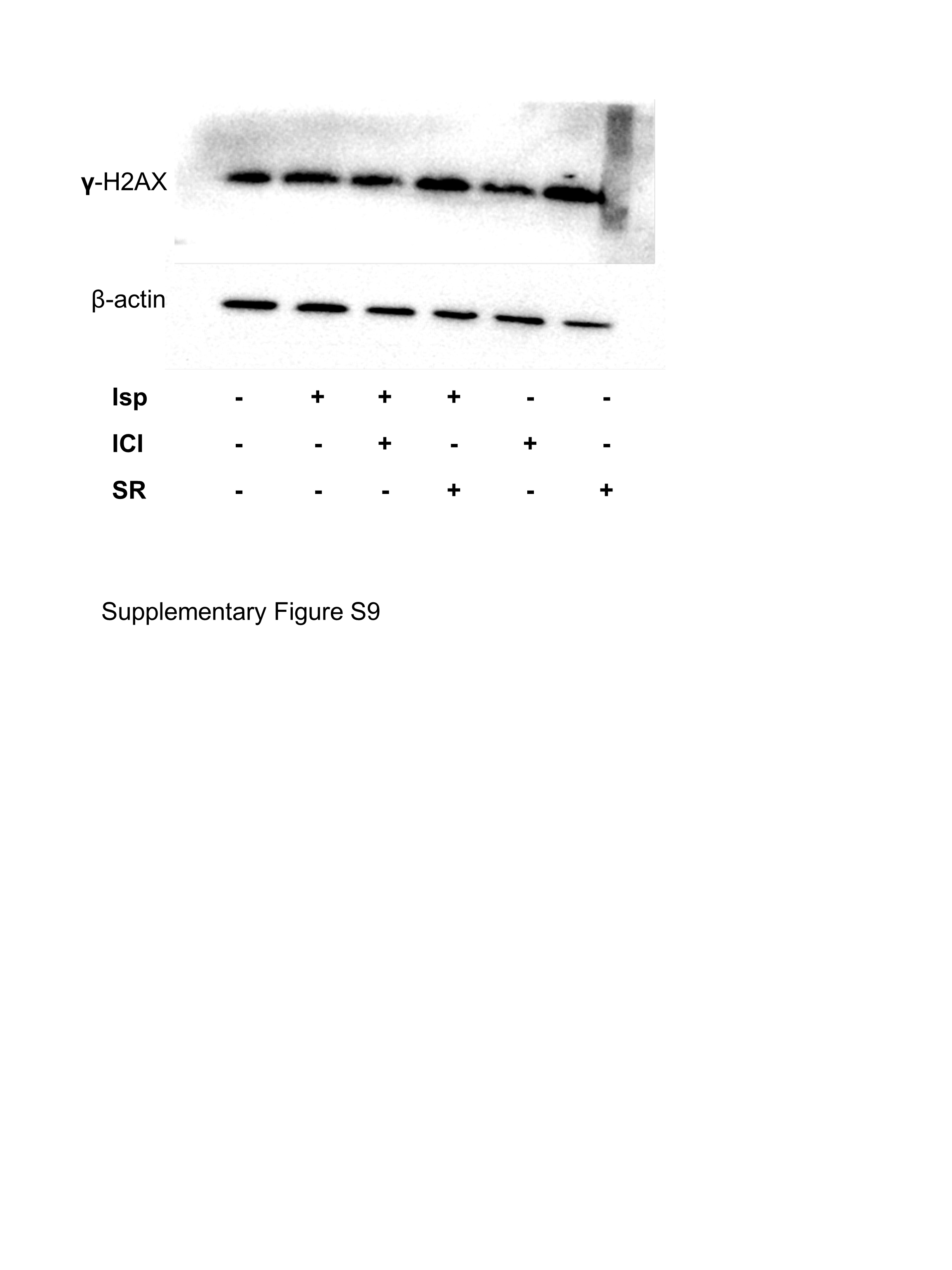


Supplementary Figure S9. Immunoblot analysis of β2 receptor-mediated γ-H2AX accumulation in ES cells. NR, non-relevant lane-occupancy loading.


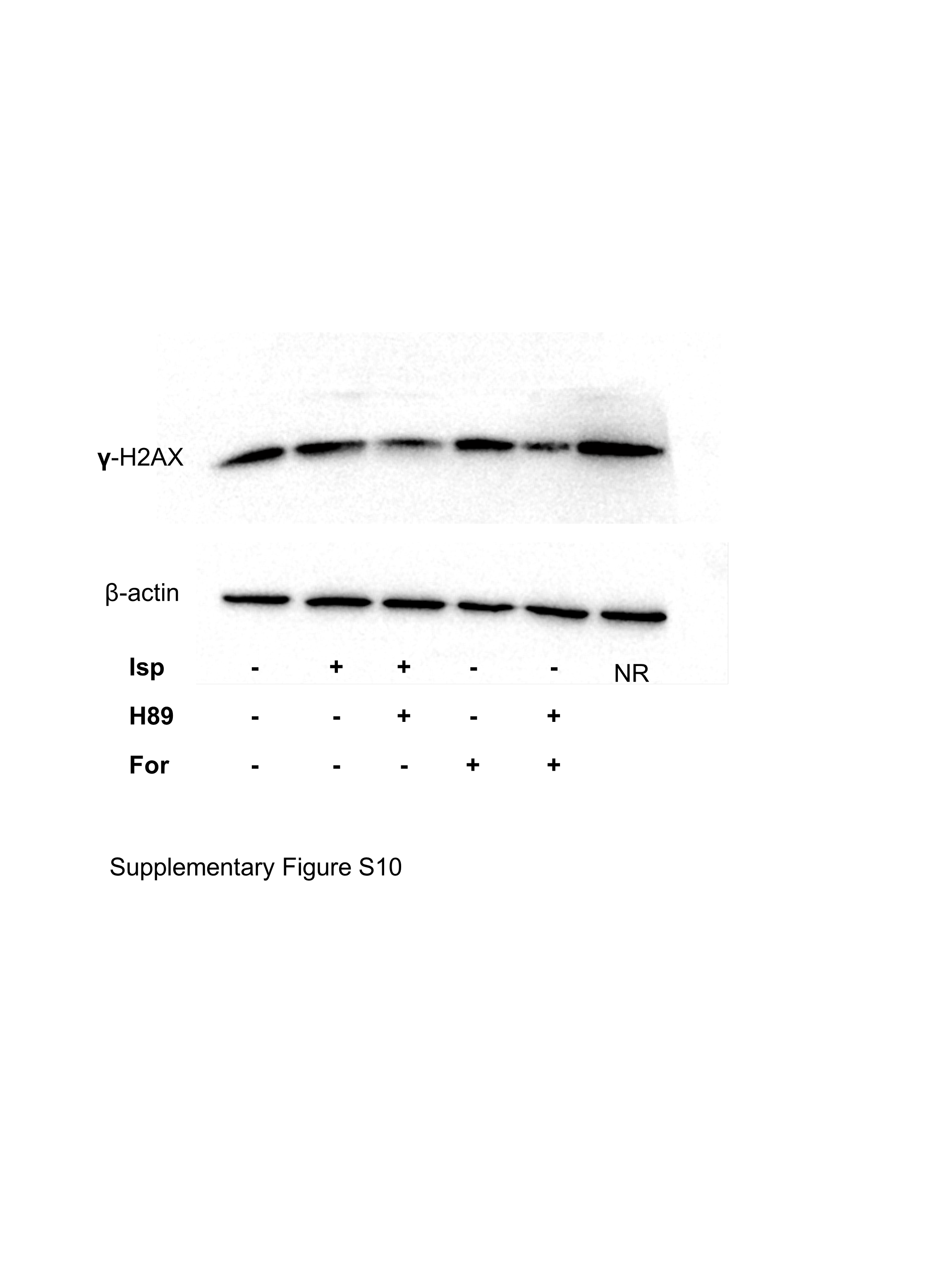


Supplementary Figure S10. Immunoblot analysis of AC/cAMP/PKA signalling-mediated γ-H2AX accumulation in ES cells. NR, non-relevant lane-occupancy loading.


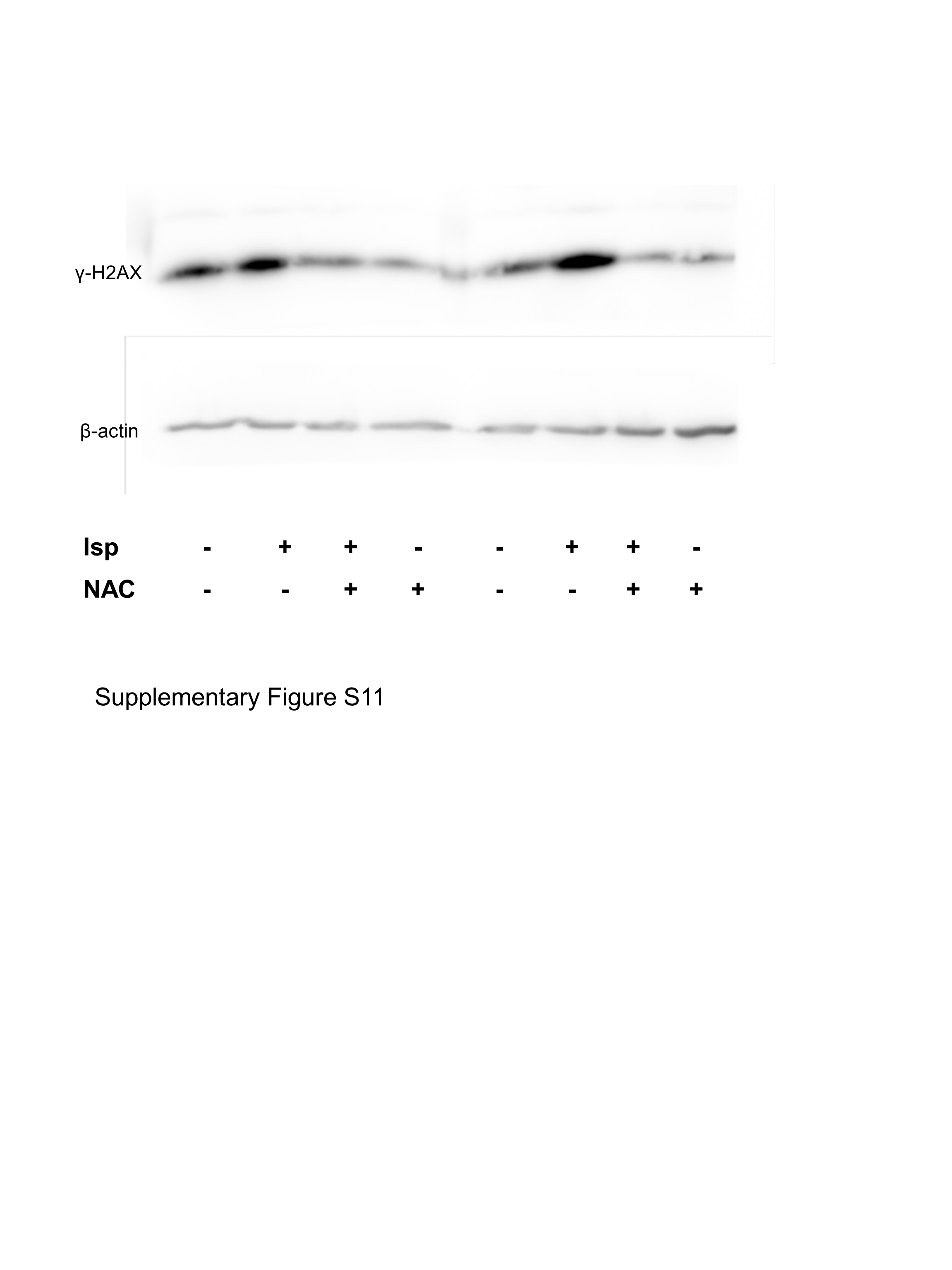


Supplementary Figure S11. Immunoblot analysis of the effect of ROS quenching on the adrenergic activation-induced γ-H2AX accumulation in ES cells.

**Supplementary Table S1. The primer sequences used for PCR.**

| β adrenergic receptor | Gene | Primer Sequences (5'-3') | Fragment (bp) |
| --- | --- | --- | --- |
| β1 | *Adrb1*_F1 | tttcgctaccagagtttgctg | 465 |
|  | *Adrb1*_R1 | gatgatgcccagtgtcttgag |  |
| β2 | *Adrb2*_F1 | ttacctcctttttgcctatcca | 485 |
|  | *Adrb2*_R1 | aagaggattgaaggcagagttg |  |
| β3 | *Adrb3*_F1 | acgccgagactacagaccata | 477 |
|  | *Adrb3*_R1 | gacgaagagcatcacaaggag |  |
| β-actin | *β-actin _F1* | gagaccttcaacaccccagc | 446 |
|  | *β-actin _R1* | ccacaggattccatacccaa |  |
